# Effects of twin-block appliance on the anatomy of pharyngeal airway passage (PAP) in class II malocclusion subjects

**DOI:** 10.1186/s40510-014-0068-3

**Published:** 2014-12-23

**Authors:** Swapnil Ghodke, Ashok Kumar Utreja, Satinder Pal Singh, Ashok Kumar Jena

**Affiliations:** Unit of Orthodontics, Oral Health Sciences Centre, Post Graduate Institute of Medical Education and Research, Sector-12, Chandigarh, India; Unit of Orthodontics, Department of Dental Surgery, All India Institute of Medical Sciences, Sijua, Dumduma, Bhubaneswar, India

**Keywords:** Class II malocclusion, Functional appliance, Twin-block appliance, Pharyngeal airway passage, Posterior pharyngeal wall thickness

## Abstract

**Background:**

The use of functional appliances for the correction of retrognathic mandible is very common in orthodontics. Similar appliances known as oral appliances are also frequently used in adults for the treatment of mild to moderate obstructive sleep apnea (OSA). Many studies have reported improvement of pharyngeal airway passage (PAP) dimensions following functional appliance therapy in children and oral appliance therapy in adults. There is only one study in the literature that discussed the effect of oral appliance therapy on posterior pharyngeal wall thickness (PPWT) among subjects with OSA. The effect of functional appliance therapy on PPWT has never been investigated. Thus the present study was conducted to evaluate the effects of twin-block appliance on pharyngeal airway passage (PAP) dimensions and posterior pharyngeal wall thickness (PPWT) in class II malocclusion subjects with retrognathic mandibles.

**Methods:**

Thirty-eight class II malocclusion subjects in the age range of 8 to 14 years with mandibular retrusion were divided into a treatment (n = 20) and control (n = 18) group. Mandibular retrusion in the treatment group subjects was corrected by twin-block appliance. The effect of twin-block appliance on PAP and PPWT dimensions were evaluated from lateral cephalograms recorded prior-to and after 6 months of appliance therapy in the treatment group subjects and the changes were compared with the changes in the control group subjects. Student’s *t*-test was used for statistical analysis; *P*-value of 0.05 was considered a statistically significant level.

**Results:**

The depth of the oropharynx was increased significantly in the treatment group subjects (*P* < 0.001) as compared to the control group subjects (*P* < 0.05). The depth of the hypopharynx increased significantly in treatment group subjects (*P* < 0.01). The PPWT at the level of the nasopharynx, oropharynx, and hypopharynx were maintained in the treatment group subjects; whereas in control group subjects, the PPWT was further reduced although the changes were not statistically significant.

**Conclusions:**

Correction of mandibular retrusion by twin-block appliance in class II malocclusion subjects increased the PAP dimensions and maintained the pre-treatment thickness of posterior pharyngeal wall.

## Background

Narrowing of the pharyngeal airway passage (PAP) and adaptations in the soft palate are common among subjects with retrognathic mandible [1,2]. Among subjects with sleep-disordered breathing (SDB), the position of the mandible is often retrognathic in relation to the cranial base [3]. As a result, the space between the cervical column and the mandibular corpus decreases and leads to a posteriorly postured tongue and soft palate, increasing the chances of impaired respiratory function during the day and possibly causing nocturnal problems like snoring, upper airway resistance syndrome, and obstructive sleep apnea (OSA) syndrome [4,5]. Till date, there is no consensus on whether the SDB in adolescents is an extension of childhood disorder or it is just a representation of early manifestation of adult form of sleep apnea, for which mandibular retrognathism is considered as one of the risk factors [3].

The use of functional appliances for the correction of retrognathic mandible is very common in orthodontics. Similar appliances known as oral appliances are also frequently used in adults for the treatment of mild to moderate OSA [6]. Many previous studies reported improvement of PAP dimensions following functional appliance therapy in children [7-14] and oral appliance therapy in adults [15-18]. Although, there is one study [19] in the literature mentioning the effect of oral appliance therapy on posterior pharyngeal wall thickness (PPWT) but there is no information in the literature mentioning the effect of functional appliance therapy on PPWT. Thus, the present study was conducted to evaluate the effect of functional appliance therapy on PPWT and PAP dimensions in class II malocclusion subjects with retrognathic mandible.

## Methods

Thirty-eight (M = 20, F = 18) consecutively treated, growing subjects in the age range of 8 to 14 years with skeletal class II malocclusion associated with mandibular retrusion were selected for this prospective longitudinal study. The subjects had skeletal class II malocclusion with normal maxilla (SNA, 79° to 84°) and retrognathic mandible (SNB ≤ 76°), Angle’s class II molar relationship bilaterally, Frankfort mandibular plane angle (FMA) in the range of 20° to 28°, minimal or no crowding or spacing in either arch, and overjet of 6 to 10 mm. Subjects with a history of orthodontic treatment, anterior open-bite, severe proclination of the anterior teeth, and any systemic disease affecting bone and general growth were excluded from the study. A written consent was obtained from each subject and the study was approved by the Institute Review Board (NK/756/MDS/1851-52).

Among 38 subjects, 20 subjects (M = 11, F = 9) in the age range of 8 to 13 years were included in treatment group and rest 18 subjects (M = 9, F = 9) in the age range of 8 to 14 years formed the control group. The mean BMI of the subjects in the treatment and control group was 16.63 ± 1.62 and 17.84 ± 1.76, respectively. The class II malocclusion in treatment group subjects was corrected by standard twin-block appliance. One-step mandibular advancement was carried out during the wax bite registration. An edge-to-edge incisor relationship with 2- to 3-mm opening between the maxillary and mandibular central incisors was maintained for all subjects. The patients were instructed to wear the appliance 24 h/day, especially during mealtimes and they were followed once in every 4 weeks. The inter-occlusal acrylic was trimmed in all subjects to allow unhindered vertical development of the mandibular buccal segments.

The control group comprised of subjects who required a phase of pre-functional therapy which included sectional fixed orthodontic appliance for the correction of mild crowding and/or rotations.

**Figure 1:**
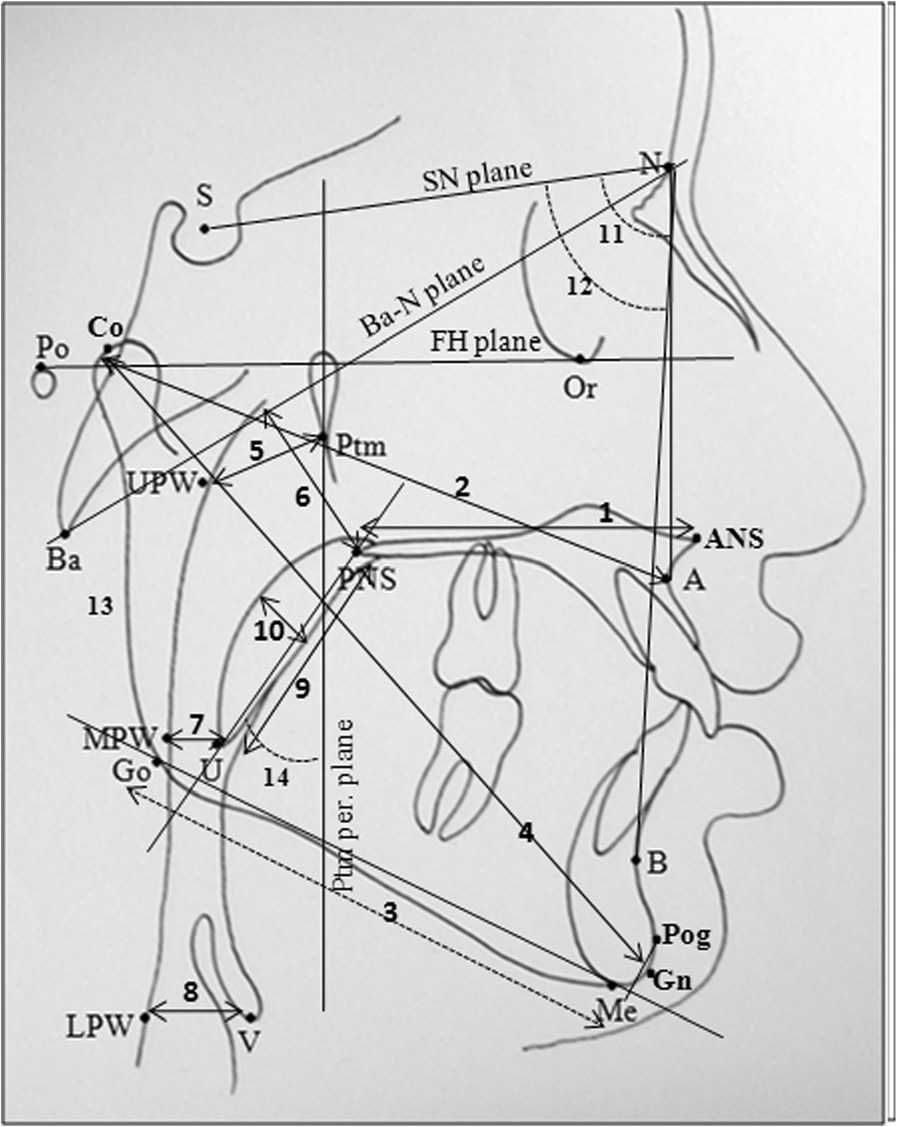
**Cephalometric landmarks, reference planes, and linear and angular parameters.** Cephalometric landmarks, reference planes, and linear and angular parameters used for evaluation of skeletal and PAP dimension changes. *Landmarks*: S, sella; N, nasion; Po, porion; Or, orbitale; Go, gonion; A, Point A; B, Point B; Pog, pogonion; Gn, gnathion; Me, menton; ANS, anterior nasal spine; PNS, posterior nasal spine; Ptm, pterygomaxillary fissure; Ba, basion; Co, condylion; U, tip of soft palate; UPW (upper pharyngeal wall), the intersection of line Ptm-Ba and posterior pharyngeal wall; MPW (middle pharyngeal wall), the intersection of perpendicular line on Ptm perpendicular from ‘U’ with posterior pharyngeal wall; V, vallecula; and LPW (lower pharyngeal wall), the intersection of perpendicular line on Ptm perpendicular from ‘V’ with posterior pharyngeal wall. *Reference planes*: SN plane, the line joining ‘S’ and ‘N’; FH plane, line joining ‘Po’ and ‘Or’; Ptm perpendicular (Ptm per), perpendicular plane on FH plane at ‘Ptm’; and Ba-N plane, line joining ‘Ba’ and ‘N.’ *Linear parameters*: 1. maxillary length (ANS-PNS); 2. effective maxillary length (Co-A); 3. mandibular length (Go-Pog⊥MP); 4. effective mandibular length (Co-Gn); 5. DNP (Ptm–UPW); 6. HNP, the shortest linear distance from PNS to Ba-N plane; 7. DOP (U–MPW); 8. DHP (V–LPW); 9. SPL (U–PNS); 10. SPT, the maximum thickness of the soft palate. *Angular parameters*: 11. SNA, angle between ‘S,’ ‘N,’ and ‘A’; 12. SNB, angle between ‘S,’ ‘N,’ and ‘B’; 13. FMA, angle between FH plane and mandibular plane (Go-Me); 14. SPI (Ptm per × PNS-U), the angle between Ptm perpendicular and the soft palate (PNS-U).

**Figure 2:**
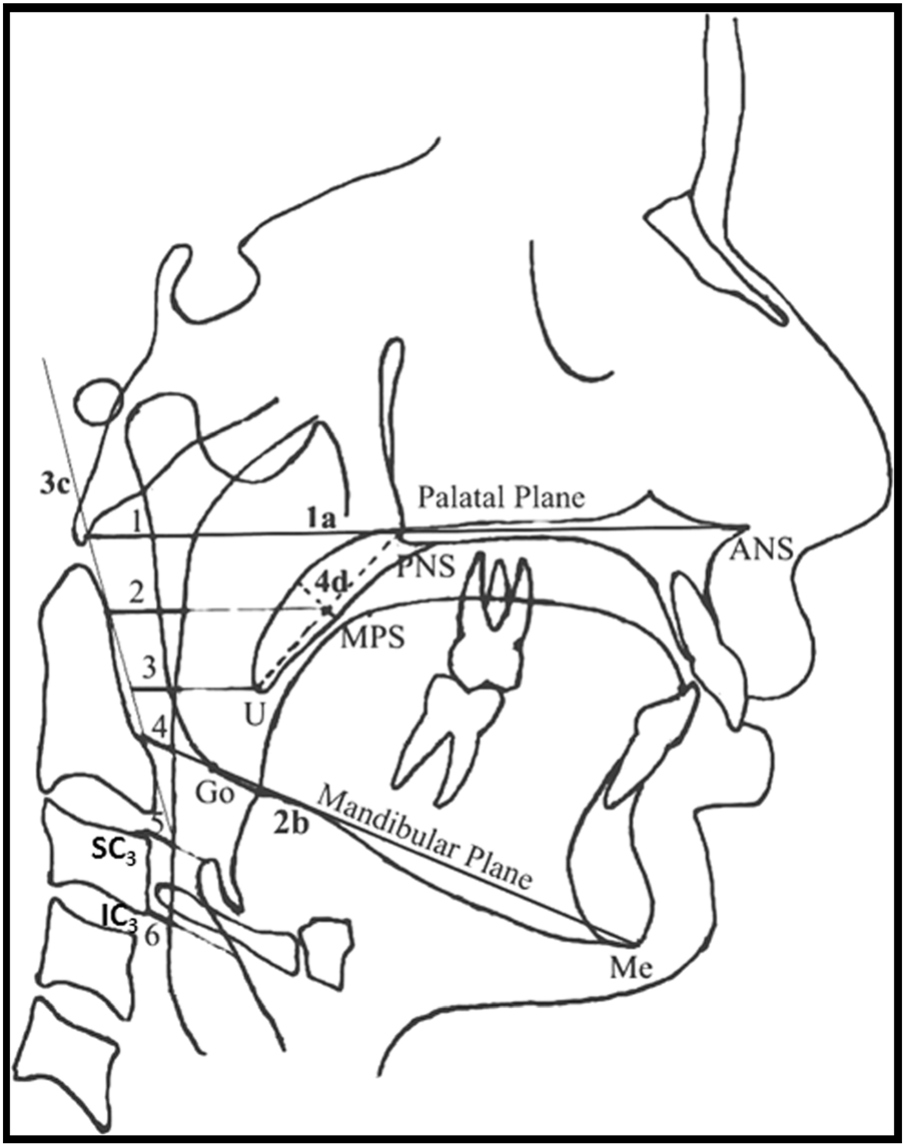
**Cephalometric landmarks, reference planes, and linear parameters used for the evaluation of PPWT change**. *Landmarks*: ANS, anterior nasal spine; PNS, posterior nasal spine; MSP, mid-point of soft palate (It is the intersection of PNS-U line and a line representing the maximum thickness of soft palate); U, tip of the soft palate; Go, gonion; Me, menton; SC_3_, superior-anterior point of C_3_ vertebra; IC_3_, inferior-anterior point of C_3_ vertebra. *Reference planes*: 1a. palatal plane (ANS-PNS); 2b. mandibular plane (Go-Me); 3c. anterior tangent to C_2_ vertebra, tangent drawn along the anterior border of C_2_ vertebra; 4d. long axis of the soft palate (PNS-U). *Linear parameters*: 1. PPWT1, the distance from the intersection point of palatal plane and posterior pharyngeal wall to the intersection point of palatal plane and anterior tangent of C_2_ vertebra; 2. PPWT2, the distance from the intersection point of line parallel to the palatal plane passing through ‘MSP’ and the posterior pharyngeal wall to the intersection point of same line extended posteriorly and anterior tangent of C_2_ vertebra. 3. PPWT3, the distance from the intersection point of line parallel to palatal plane passing through the ’U’ and the posterior pharyngeal wall to the intersection point of same line extended posteriorly and anterior tangent of C_2_ vertebra; 4. PPWT4, the distance from the intersection point of the mandibular plane and posterior pharyngeal wall to the intersection point of the mandibular plane and anterior tangent of C_2_ cervical vertebra; 5. PPWT5, the distance from the intersection point of line parallel to the mandibular plane passing through the superior-anterior point of C_3_ vertebra and the posterior pharyngeal wall to superior-anterior point of C_3_ vertebra; 6. PPWT6, the distance from the intersection point of line parallel to mandibular plane passing through the inferior-anterior point of C_3_ vertebra and the posterior pharyngeal wall to inferior-anterior point of C_3_ cervical vertebra.

The skeletal, PAP dimension, and PPWT changes were evaluated from lateral cephalograms. Lateral cephalograms with teeth in occlusion were obtained for all subjects before the start of treatment (T_0_) and after a follow-up period of approximately 6 months (T_1_) in treatment subjects and at the beginning (T_0_) and after 6 months (T_1_) of observation in control subjects. While recording the lateral cephalograms, patients were placed in the standing position with FH plane parallel to the floor and teeth in centric occlusion. The head of the patient was erect. The cephalogram was exposed at the end-expiration phase of the respiration. Subjects were instructed not to move their head and tongue and not to swallow during cephalogram exposure. All cephalograms were recorded in the same machine with same exposure parameters. The dimensions of PAP were determined according to the method described by Jena et al. [2] and the PPWT was determined according to the method described by Joseph et al. (1998) [20]. All lateral cephalograms were traced manually. Various landmarks, reference planes, and linear and angular parameters used for the evaluation of skeletal and PAP dimension changes are described in Figure 1; and various landmarks, reference planes, and linear parameters used for the evaluation of PPWT change are described in Figure 2. All the variables were measured thrice and their mean was subjected for statistical analysis. The assessment of intra-observer variability and reproducibility of landmark location and measurement errors was analyzed by retracing the 10% randomly selected cephalograms after a gap of 15 days. The method error was calculated according to Dahlberg’s formula [21]. The reliability of measurements is described in Table 1.

**Table 1:** **The reliability for the measurement of various cephalometric variables**

**Parameter**	**Method error**	**Mode of variance**	**Reliability**
SNA (°)	0.52	3.66	0.92
Maxillary length (mm)	0.68	19.84	0.98
Effective maxillary length (mm)	0.46	21.15	0.99
SNB (°)	0.45	2.77	0.93
Mandibular length (mm)	0.49	19.44	0.99
Effective mandibular length (mm)	0.84	37.04	0.98
FMA(°)	0.35	14.23	0.99
Depth of the nasopharynx [DNP] (mm)	0.54	19.40	0.99
Height of the nasopharynx [HNP] (mm)	0.61	3.61	0.90
Depth of the oropharynx [DOP] (mm)	0.57	9.06	0.96
Depth of the hypopharynx [DHP] (mm)	0.54	12.41	0.98
Soft palate length [SPL] (mm)	1.00	11.26	0.91
Soft palate thickness [SPT] (mm)	0.38	1.51	0.90
Soft palate inclination [SPI] (°)	1.67	32.01	0.91
PPWT 1 at nasopharyngeal space 1 (mm)	0.69	9.82	0.95
PPWT 2 at nasopharyngeal space 2 (mm)	0.65	7.36	0.94
PPWT 3 at oropharyngeal space 1 (mm)	0.60	1.57	0.77
PPWT 4 at oropharyngeal space 2 (mm)	0.53	6.36	0.96
PPWT 5 at hypopharyngeal space 1 (mm)	0.29	0.77	0.89
PPWT 6 at hypopharyngeal space 2 (mm)	0.30	1.15	0.92

### Statistical analysis

The statistical analysis was carried out using SPSS software (version-16.0). Descriptive statistics were used. Shapiro-Wilk test was used to examine the normality of the data. The significant changes within the group (pre- and post-treatment/post-follow-up values) were determined by paired ‘*t*’-test and the mean differences among the groups were compared by Student ‘*t*’-test. The *P*-value of 0.05 was considered as level of significance.

## Results

The mean age of the subjects at the beginning of the study in treatment and control group subjects was 10.90 ± 1.48 years and 10.94 ± 1.86 years, respectively. The mean duration of follow-up of subjects in treatment and control group was 244.63 ± 35.58 days and 222.80 ± 32.91 days, respectively.

The skeletal changes in the treatment and control group subjects are described in Table 2. The change in effective maxillary length in treatment group subjects was significantly less as compared to control group subjects (P < 0.01). The change in sagittal position of the mandible (SNB angle) was significantly more in treatment group subjects compared to the control group subjects (*P* < 0.001). The change in the length of the mandible was significantly more in treatment group subjects as compared to control group subjects (*P* < 0.01). The FMA increased significantly in treatment group subjects (*P* < 0.01).

**Table 2:** **Changes in the skeletal tissue among treatment and control group subjects**

**Variables**	**Groups**						**Comparison of mean difference among treatment and control groups (** ***P*** **-value)**
	**Treatment group**			**Control group**			
	**Pre-treatment (T**_**0**_ **)**	**6 months post-treatment (T**_**1**_ **)**	**Significance (** ***P*** **-value)**	**Pre-follow-up (T**_**0**_ **)**	**6 months post-follow-up (T**_**1**_ **)**	**Significance (** ***P*** **-value)**	
	**Mean ± SD**	**Mean ± SD**		**Mean ± SD**	**Mean ± SD**		
SNA (°)	81.10 ± 1.80	81.43 ± 1.86	0.097^NS^	81.28 ± 1.71	81.47 ± 1.87	0.049*	0.547 ^NS^
Maxillary length (mm) (ANS-PNS)	48.21 ± 3.56	48.70 ± 3.43	0.089^NS^	48.50 ± 3.46	49.17 ± 3.50	0.000***	0.275^NS^
Effective maxillary length (mm) (Co-A)	80.27 ± 5.37	80.56 ± 5.14	0.303^NS^	81.19 ± 5.41	82.36 ± 5.04	0.002**	0.040*
SNB (°)	73.95 ± 1.91	75.85 ± 1.66	0.000***	73.94 ± 1.73	74.33 ± 1.75	0.069^NS^	0.000***
Mandibular length (mm) (Go-Pog ⊥ MP)	63.21 ± 3.99	64.39 ± 4.14	0.000***	63.35 ± 4.09	63.82 ± 4.36	0.135^NS^	0.004**
Effective mandibular length (mm) (Co-Gn)	96.55 ± 5.23	99.90 ± 5.54	0.000***	96.39 ± 6.84	98.52 ± 6.42	0.002**	0.007**
FMA(°)	25.48 ± 2.15	26.55 ± 2.44	0.000***	24.03 ± 2.76	24.22 ± 2.71	0.0130^NS^	0.004**

SD indicates standard deviation; NS, nonsignificant; *P < .05; **P < .01; ***P < .001.

SNA, angle between ‘S,’ ‘N,’ and ‘A’; it represents the antero-posterior position of the maxilla in relation to the anterior cranial base; maxillary length, the linear distance between ‘ANS’ and ‘PNS’ points; effective maxillary length, the linear distance between ‘Co’ and ‘point A’; SNB, angle between ‘S,’ ‘N,’ and ‘B’; it represents the antero-posterior position of the mandible in relation to the anterior cranial base; mandibular length, the linear distance between ‘Go’ and the intersection of the perpendicular drawn from ‘Pog’ on mandibular plane (Go-Me); effective mandibular length, the linear distance between the ‘Co’ and ‘Gn’; FMA indicates Frankfort mandibular plane angle.

The PAP dimension changes in the treatment and control group subjects are described in Table 3. The DOP improved by 1.54 mm in treatment group subjects (*P* < 0.001) where as it was increased by 0.89 mm (*P* < 0.05) in control group subjects. The improvement of DOP among the treatment group subjects was significantly more compared to the control group subjects (*P* < 0.05). The DHP was improved significantly in treatment group subjects (*P* < 0.01). The SPL was decreased in treatment group subjects whereas it increased marginally in control group subjects. The SPT was increased in the treatment group subjects, but it was decreased in control group subjects. The SPI was decreased significantly (*P* < 0.05) in treatment group subjects where as it increased in control group subjects. The length and thickness of the soft palate in treatment group subjects were improved compared to control group subjects but the differences were not significant. The inclination of the soft palate decreased significantly in treatment group subjects (*P* < 0.05) and the difference between the treatment and control group was statistically significant (P < 0.05).

**Table 3:** **The changes in the pharyngeal airway passage (PAP) dimensions among treatment and control group subjects**

**Variables**	**Groups**						**Comparison of mean difference among treatment and control groups (P-value)**
	**Treatment Group**			**Control Group**			
	**Pre-treatment (T**_**0**_ **)**	**6-months Post-treatment (T**_**1**_ **)**	**Significance (P-value)**	**Pre-follow-up (T**_**0**_ **)**	**6-months Post-follow-up (T**_**1**_ **)**	**Significance (P-value)**	
	**Mean ± SD**	**Mean ± SD**		**Mean ± SD**	**Mean ± SD**		
DNP (mm) (Ptm-UPW)	12.84 ± 5.77	13.76 ± 5.17	0.254^NS^	12.01 ± 5.78	13.04 ± 5.15	0.065^NS^	0.911^NS^
HNP (mm) (PNS to Ba-N plane)	21.76 ± 1.78	21.93 ± 2.22	0.471^NS^	21.25 ± 1.85	21.78 ± 2.20	0.034*	0.258^NS^
DOP (mm) (U-MPW)	9.19 ± 2.03	10.73 ± 2.45	0.000***	7.81 ± 2.13	8.70 ± 1.80	0.010*	0.013*
DHP (mm) (V-LPW)	12.53 ± 2.80	14.30 ± 2.99	0.005**	12.84 ± 2.12	13.21 ± 2.15	0.516^NS^	0.081^NS^
SPL (mm) (U-PNS)	30.56 ± 3.41	30.01 ± 3.25	0.136^NS^	30.78 ± 2.85	30.88 ± 3.25	0.740^NS^	0.365^NS^
SPT (mm) (Maximum thickness of the soft palate)	7.24 ± 1.15	7.48 ± 1.02	0.135^NS^	7.11 ± 0.76	7.09 ± 0.96	0.845^NS^	0.287^NS^
SPI (°) (Ptm per × PNS-U)	46.75 ± 4.72	44.22 ± 3.90	0.019*	42.35 ± 5.40	44.30 ± 4.82	0.068^NS^	0.045*

SD indicates standard deviation; NS, nonsignificant; *P < .05; **P < .01; ***P < 0.001.

DNP, depth of the nasopharynx; HNP, height of the nasopharynx; DOP, depth of the oropharynx; DHP, depth of the hypopharynx; SPL, soft palate length; SPT, soft palate thickness; and SPI, soft palate inclination.

The changes in the PPWT in treatment and control group subjects are described in Table 4. The PPWT at the region of the nasopharynx (PPWT1 and PPWT2), oropharynx (PPWT3 and PPWT4), and hypopharynx (PPWT5 and PPWT6) were maintained in treatment group subjects whereas the PPWT at various regions of the upper airway further decreased in control group subjects but the difference between two groups was not statistically significant.

**Table 4:** **The changes in the posterior pharyngeal wall thickness (PPWT) among treatment and control group subjects**

**Variables**	**Groups**						**Comparison of mean difference among treatment and control groups (** ***P*** **-value)**
	**Treatment group**			**Control group**			
	**Pre-treatment (T**_**0**_ **)**	**6 months post-treatment (T**_**1**_ **)**	**Significance (** ***P*** **-value)**	**Pre-follow-up (T**_**0**_ **)**	**6 months Post-follow-up (T**_**1**_ **)**	**Significance (** ***P*** **-value)**	
	**Mean ± SD**	**Mean ± SD**		**Mean ± SD**	**Mean ± SD**		
PPWT 1 (mm)	15.68 ± 4.15	15.97 ± 4.18	0.646^NS^	16.06 ± 4.56	14.54 ± 3.36	0.015*	0.226^NS^
PPWT 2 (mm)	11.02 ± 3.19	11.02 ± 3.00	0.991^NS^	10.78 ± 2.99	10.23 ± 2.67	0.217^NS^	0.370^NS^
PPWT 3 (mm)	4.66 ± 1.41	4.84 ± 1.83	0.521^NS^	5.52 ± 1.65	5.00 ± 1.84	0.209^NS^	0.150^NS^
PPWT 4 (mm)	4.38 ± 2.41	4.74 ± 2.27	0.338^NS^	5.09 ± 2.84	4.65 ± 2.72	0.239^NS^	0.875^NS^
PPWT 5 (mm)	4.10 ± 1.04	4.10 ± 1.07	0.988^NS^	4.45 ± 1.14	4.32 ± 1.22	0.534^NS^	0.684^NS^
PPWT 6 (mm)	3.28 ± 0.94	3.52 ± 0.84	0.242^NS^	3.65 ± 1.05	3.44 ± 0.83	0.361^NS^	0.896^NS^

SD indicates standard deviation; NS, non-significant; *p<0.05.

PPWT1, posterior pharyngeal wall thickness at nasopharyngeal space 1; PPWT2, posterior pharyngeal wall thickness at nasopharyngeal space 2; PPWT3, posterior pharyngeal wall thickness at oro-pharyngeal space 1; PPWT4, posterior pharyngeal wall thickness at oropharyngeal space 2; PPWT5, posterior pharyngeal wall thickness at hypopharyngeal space 1; PPWT6, posterior pharyngeal wall thickness at hypopharyngeal space 2.

## Discussion

Small PAP dimension and anatomical adaptation of the soft palate are common features in subjects with retrognathic mandible [4,5,22]. Correction of mandibular retrognathism by functional appliances improves the dimensions of the upper airway [7-14]. Although lateral cephalograms are not ideal for the airway analysis, yet its use is an established tool [23]. Reproducibility of airway dimensions on lateral cephalograms was also found as highly accurate [24]. Although 3D imaging would be an appropriate method for the evaluation of PAP dimension, the technique is not available in all centers and has the risk of relatively high radiation dose. Therefore, the conventional lateral cephalogram still remains as a valuable and reliable diagnostic tool in numerous airway studies.

The present study showed that the sagittal jaw relationship improved significantly in treatment group subjects. When the mandible was postured forward by the twin-block appliance, a reciprocal force acted distally on the maxilla, restricting its forward growth and stimulating the forward mandibular growth. Many previous studies also reported similar observation following twin-block therapy [25-29].

In our class II controls, the PAP dimension change was very minimum. Hänggi et al. [8] also reported no significant change in the PAP dimensions during adolescence. However, we observed significant improvements in the depth of the oropharynx and hypopharynx, and inclination of the soft palate following correction of mandibular retrusion in class II malocclusion subjects. The backward position of the tongue in subjects with retrognathic mandible pushed the soft palate posterior and decreased the dimension of the upper airway [2]. When the mandible was displaced anteriorly by the twin-block appliance, it influenced the position of the hyoid bone and consequently the position of the tongue and thus improved the morphology of the upper airway [30]. Recently, Jena et al. [14] also reported increase in the PAP dimension following twin-block therapy among subjects with retrognathic mandible. Schutz et al. [13] found that after class II correction, the anterior displacement of the mandible and the hyoid bone caused an anterior traction of the tongue, which increased the posterior airway space by 3.2 mm and reduced the airway resistance. However in contrast to our study, Fastuca et al. [31] reported no improvement in the oropharyngeal airway dimensions following mandibular displacement after maxillary expansion in growing patients.

The benefits of oral appliance therapy on upper airway dimension in OSA patients are well established [15-18]. Similar benefits are also produced by various functional appliances [7-14]. Few authors have investigated the thickness of the posterior pharyngeal wall in OSA subjects [32-34] and the effects of oral appliances on the PPWT [19]. The PPWT in subjects with OSA has been reported to be more compared to the normal subjects [34] and the oral appliance therapy had no significant effect on PPWT [19]. However, the present study showed that the PPWT at the nasopharynx, oropharynx, and hypopharynx level was maintained in treatment group subjects and it further decreased in thickness in the control group subjects. This observation showed that the upper airway tried to maintain its patency by reducing the thickness of the posterior pharyngeal wall as a compensatory mechanism among subjects with retrognathic mandible who did not receive any treatment. As the sagittal dimension of PAP was increased secondary to the forward posture of the tongue caused by anterior relocation of the mandible by twin-block appliance, it reduced the compensatory adaptation in the PPWT and as a result, the thickness got marginally increased. However, Cozza et al. (2008) reported that the use of oral appliances in OSA patients had no effect on the thickness of the posterior pharyngeal wall, but it did produce a significant expansion by 13% in the areas most involved in the collapse [19].

Thus, the present study showed that there is a positive impact of twin-block appliance therapy on the PAP dimension and PPWT. The literature also supports that the changes in the PAP dimension following functional appliance therapy are maintained in long term [8,35]. Thus, class II correction by twin-block appliance during childhood might help to eliminate the adaptive changes in the upper airway and predisposing factors to OSA, thus decreasing the risk of OSA development in adulthood.

## Conclusions

The following conclusions were drawn from the present study:

1. Correction of mandibular retrusion in class II malocclusion subjects by twin-block appliance increased the sagittal dimension of the oropharynx and hypopharynx.
2. The length, thickness, and inclination of the soft palate improved following correction of mandibular retrusion in class II malocclusion subjects.
3. The correction of mandibular retrusion by twin-block appliance in class II malocclusion subjects had no significant effect on the posterior pharyngeal wall thickness.
